# Pilot study of fluvoxamine treatment for climacteric symptoms in Japanese women

**DOI:** 10.1186/1751-0759-1-12

**Published:** 2007-06-05

**Authors:** Akira Oishi, Yoshiko Mochizuki, Reiko Otsu, Noriyuki Inaba

**Affiliations:** 1 Department of Obstetrics and Gynecology, Dokkyo University School of Medicine, Mibu, Tochigi, Japan

## Abstract

**Background:**

Selective serotonin-reuptake inhibitors (SSRIs) are commonly prescribed for the treatment of depression and can be used as nonhormonal alternatives to manage hot flashes for women with a history of breast cancer and unable to take hormone replacement therapy. There are, however, few reports on the efficacy of SSRIs for the treatment of natural postmenopausal climacteric symptoms. In this pilot study, we evaluate the SSRI, fluvoxamine, for controlling climacteric symptoms and vasomotor symptoms, in particular.

**Methods:**

Twenty-two patients were enrolled from our hospital. All were orally administered fluvoxamine (50 mg daily). Climacteric and depressive symptoms were assessed using simple menopausal index (SMI) and self-rating questionnaire for depression (SRQ-D), respectively, at baseline, and at 2 and 6 weeks post-treatment.

**Results:**

Six weeks following drug administration, neither the SRQ-D nor SMI scores significantly decreased compared to baseline. The mean levels of vasomotor symptoms and mental symptoms decreased significantly following fluvoxamine administration, while skeletal muscle symptom scores did not.

**Conclusion:**

We were able to demonstrate that fluvoxamine was effective in treating not only depressive moods in climacteric symptoms but also the associated vasomotor symptoms. There are several limitations to this preliminary study. Future controlled studies are needed to further evaluate the efficacy of fluvoxamine for climacteric disturbances.

## Background

Perimenopause is an estrogen-deficient state often associated with vasomotor symptoms including hot flashes, night sweats, and vaginal dryness. Hormone replacement therapy (HRT) is effective in reducing the incidence and severity of vasomotor symptoms by as much as 80% [1]. For quite some time, HRT has been the gold standard treatment for climacteric symptoms. However, despite the robust evidence supporting its use for hot flashes, recent findings from the Women's Health Initiative (WHI) Study suggest that combined conjugated equine estrogen and progestin therapy cannot be recommended to most women, as it increases the risks for coronary heart and thromboembolic disease [2,3]. Therefore, nonhormonal alternative treatments are needed.

Serotonin levels in the brain of postmenopausal women are lower than in women who have not yet entered menopause [4]. Thus, it is thought that the serotonergic system plays a major role in menopause.

Selective serotonin-reuptake inhibitors (SSRIs) are commonly prescribed for the treatment of depression. Several studies have found SSRIs improve the incidence of hot flashes and other menopausal symptoms in women with breast cancer [5,6]. Stearns *et al*. evaluated that the SSRI paroxetine, may be an effective and acceptable alternative to HRT and other therapies in treating menopausal hot flash symptoms [7]. In 1999, fluvoxamine was the first antidepressant SSRI introduced in Japan. It is an effective treatment for postpartum depression [8]. To date, there are no reports of fluvoxamine used as an effective monotherapy for vasomotor symptoms such as hot flashes. The aim of this study was to investigate whether fluvoxamine is an effective treatment for these spontaneous climacteric symptoms.

## Methods

Twenty-two patients who presented to our climacteric outpatient clinic on Dokkyo University School of Medicine were enrolled. They were classified as either perimenopausal (N = 8) and postmenopausal (N = 14). Perimenopausal status was defined as having irregular menstrual cycles (fewer than six menstrual cycles per year) and follicle-stimulating hormone (FSH) levels greater than 20 IU/liter, documenting declining ovarian function. Postmenopausal status was defined as being amenorrheal for 12 months or more, or having had a bilateral oophorectomy. Clinical climacteric and depressive symptoms were assessed at study entry using the simple menopausal index (SMI) and Self-rating Questionnaire for Depression (SRQ-D).

SMI, a questionnaire with the ten most common climacteric symptoms for Japanese perimenopausal women, was used to identify the symptoms and to determine severity before treatment. A full score of SMI is one hundred. SMI scores of each group were classified according to three subgroups of climacteric symptoms; vasomotor, mental, and skeletal muscle, and then assessed according to severity (severe, moderate, mild, free) (Table 1). We used the SRQ-D to evaluate depressive moods. A full score of SRQ-D is 36 points. Those women with an SRQ-D score >12 were regarded as having depressive symptoms. The SRQ-D is suitable for evaluating masked depression; it includes many questions concerning depression-related physical symptoms, where a score of ≧11 indicates possible depression [9,10].

**Table 1:** Items used for the simple menopausal index (SMI)

	**severe**	**moderate**	**mild**	**free**
1. Hot flushes	10	6	3	0
2. Episodic sweating	10	6	3	0
3. Cold limbs	14	9	5	0
4. Heart discomfort	12	8	4	0
5. Sleeping disorders	14	9	5	0
6. Irritability	12	8	4	0
7. Depression	7	5	3	0
8. Headache	7	5	3	0
9. Fatigue	7	4	2	0
10. Shoulder stiffness/lumbago	7	5	3	0

SMI scores of climacteric symptoms for the three subgroups. Each group composed of vasomotor symptoms (No. 1 to 4), mental symptoms (No. 5 to 8), and skeletal muscle symptoms (No. 9 and 10).

All patients were administered fluvoxamine (DEPROMEL^®^; Meiji Seika Kaisha, Japan) 50 mg daily orally. Climacteric and depressive symptom assessments were repeated 2 and 6 weeks after treatment using the SMI and SRQ-D. Informed consent was obtained from all patients, and the research procedure was approved by the local ethics committee. Exclusion criteria included medical illness, use of psychoactive drugs within 3 months before assessment, abnormalities on screening ultrasounds, and clinical contraindications to antidepressant therapy.

**Figure 1:**
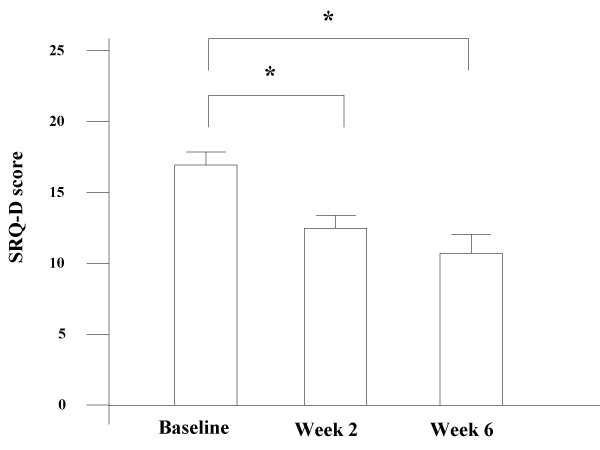
**Changes in the SRQ-D scores**. Changes in the SRQ-D scores at baseline, after 2, and 6 weeks of fluvoxamine treatment. Values are expressed as mean ± SE, where *P < 0.05 vs baseline.

**Figure 2:**
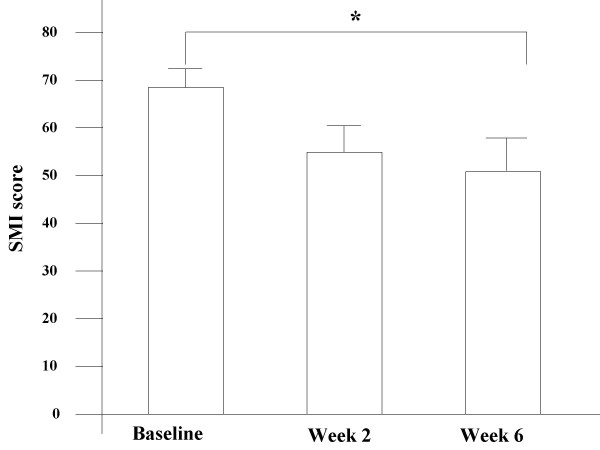
**A – Changes in the simple menopausal index (SMI)**. Changes in the simple menopausal index (SMI) at baseline, after 2, and 6 weeks of treatment with fluvoxamine, where *P < 0.05 vs baseline. **B – Percent of change from baseline among subgroups according to SMI **Percent of change from baseline among subgroups according to SMI at 6 weeks of fluvoxamine treatment, where *P < 0.05 vs baseline; NS: No significant change.

All data are expressed as mean values ± SE. Statistical analysis was performed where appropriate by using SPSS software version 8.0 (SPSS Inc., Chicago, IL). Analytical tests were repeated measures ANOVA (Figure 1, Figure 2A), and paired t-test (Figure 2B). *P*-values less than 0.05 were considered statistically significant.

## Results

### Medical characteristics

A total of 22 women participated in the pilot trial, where the mean age was 52.9 ± 7.6 years old. All women were evaluated for safety and tolerability. Pretreatment SRQ-D scores were 17.3 ± 1.3 and SMI scores were 68.5 ± 4.2. Additional medical information for the study participants are listed in Table 2. Four women prescribed fluvoxamine discontinued drug therapy due to adverse effects (nausea 2, mouth dryness 1, dizziness 1).

**Table 2:** Characteristics of the patients

Number of patients	22
Peri menopause	8
Post menopause	14
Age (yr)	52.9 ± 7.6
BMI	22.6 ± 2.7
FSH (mIU/ml)	80.8 ± 42.4
SRQ-D score	17.3 ± 1.3
SMI score	68.5 ± 4.2

Characteristics of patients (N = 22) receiving fluvoxamine at start of trial. Shown are mean ± SE.

### Reduction of depressive symptoms

Depressive symptoms were moderately severe at baseline (median SRQ-D scores = 17.3 ± 1.3). SRQ-D scores after 2 weeks of treatment were significantly decreased compared with that at baseline. Similarly, fluvoxamine improved SRQ-D scores after 6 weeks (*P *< 0.05, Figure 1). All patients had a SRQ-D score >13 at the start of treatment, and five patients had decreased SRQ-D scores (SRQ-D <11) under normalization after 6 weeks of treatment.

### Reduction of SMI scores

SMI scores expressed clinically climacteric symptoms, including mental symptoms. Following fluvoxamine administration at week 6, SMI scores declined to 50.1 ± 7.1 from baseline (*P *< 0.05, Figure 2A). Furthermore, we performed a detailed investigation for SMI score. SMI scores were divided into three subgroups of climacteric symptoms, vasomotor, mental and skeletal muscle symptoms. The mean levels of vasomotor and mental symptoms decreased significantly following fluvoxamine administration (*P *< 0.05, Figure 2B). However, there was no statistically significant difference between pre and post treatment of skeletal muscle symptoms (lumbago and general fatigue).

## Discussion

In this study we demonstrated the effectiveness of fluvoxamine on climacteric symptoms. Climacteric symptoms occur in association with menopause. Drastic changes in hormone levels occur during perimenopause [11]. It was reasonable to assume that climacteric symptoms are caused by low levels of sex hormones. Most reports show that HRT has a beneficial effect on climacteric symptoms [12,13]. Vasomotor symptoms, in particular hot flashes, are the most common complaint among women entering menopause. The physiology behind vasomotor symptoms is not fully understood, although the main underlying cause is thought to be a disturbance in normal thermoregulatory function. Given that there are strong interactions with estrogen reduction and vasomotor symptoms, some menopausal women would benefit from estrogen to alleviate their vasomotor symptoms.

The WHI single-arm study, however, showed HRT increased the occurrence of breast cancer and myocardial infarction in comparison with placebo control [2]. Therefore, nonhormonal treatments include alternative treatments for menopause-related symptoms, and ultimately improvements in quality of life. It is possible SSRIs can be used to treat vasomotor symptoms since there is a strong interaction between estrogen and the serotonergic system [14]. Recent clinical studies of venlafaxine and fluoxetine in women with a prior history of breast cancer have suggested that certain antidepressants, with the ability to inhibit serotonin reuptake, may significantly reduce the vasomotor symptoms associated with menopause [5,6,15]. The SSRI fluvoxamine is an effective antidepressant that acts by facilitating serotonergic neurotransmission [16,17]. In our fluvoxamine trials, the SSRI showed beneficial effects; that is an improvement in vasomotor and mental symptoms appeared statistically significant in accordance to other SSRI reports.

HRT's inability to deal with symptoms of general fatigue and lumbago has been documented [18,19]. As Weitzner *et al*. demonstrated most SSRI treatment for breast cancer in women with hot flashes had significant improvement in their general levels of fatigue, as well as their emotional and mental levels of fatigue [20]. Our results suggest that fluvoxamine treatment was unsuccessful (p > 0.05) for general fatigue and lumbago accompanied by climacteric symptoms, potentially due to the limited power of statistical analyses from the small sample size and dose. Also, it is possible the SSRIs may be ineffectual for skeletal muscle symptoms; given the high frequency of general fatigue and lumbago, and that SSRIs are insufficient in relieving these symptoms.

Although this study is limited by its uncontrolled design and small sample size, our data suggests fluvoxamine, along with other SSRIs, decreases the level of depressive climacteric disturbances. The pathophysiology of menopausal mood disorders remains to be determined; however, sex specificity suggests that hormones have an impact on the incidence of depression [21]. The absence of estrogen in the brain during menopause results in decreased release of serotonin from the neurons and a negative mood state, including irritability and depression [22]. As a result, medications such as SSRIs cause serotonin to remain in the synapse longer, thus leading to amelioration in negative mood states caused by a decrease in estrogen [23].

## Conclusion

In summary, although these data are preliminary and there are several limitations to this study, we were able to demonstrate that the SSRI, fluvoxamine, was effective in treating depressive moods associated with menopause. Our data also suggests improvements in vasomotor and mental symptom. Future studies using larger groups of patients are necessary to assess the relative effectiveness and morbidity of this treatment option.

## Competing interests

The author(s) declare that they have no competing interests.

## Authors' contributions

AO conceived of this study, and performed the statistical analysis and drafted the manuscript. YM conceived of the study, and participated in its design and coordination and helped to draft the manuscript. RO helped the statistical analysis. NI conceived of the study, and participated in its design and coordination. All authors read and approved the final manuscript. All authors contributed equally to this work.
